# Human-Centric Cognitive State Recognition Using Physiological Signals: A Systematic Review of Machine Learning Strategies Across Application Domains

**DOI:** 10.3390/s25134207

**Published:** 2025-07-05

**Authors:** Kaizhe Jin, Adrian Rubio-Solis, Ravi Naik, Daniel Leff, James Kinross, George Mylonas

**Affiliations:** 1 Hamlyn Centre for Robotic Surgery, Institute of Global Health Innovation, Imperial College London, London SW7 2AZ, UK; k.jin20@imperial.ac.uk (K.J.); a.rubio-solis@imperial.ac.uk (A.R.-S.); ravi.naik15@imperial.ac.uk (R.N.); d.leff@imperial.ac.uk (D.L.); 2 Department of Surgery & Cancer, Imperial College London, London SW7 2AZ, UK; j.kinross@imperial.ac.uk

**Keywords:** cognitive state recognition, deep learning, human-centric AI, physiological signals

## Abstract

This systematic review analyses advancements in cognitive state recognition from 2010 to early 2024, evaluating 405 relevant articles from an initial pool of 2398 records identified through five databases: Scopus, Engineering Village, Web of Science, IEEE Xplore, and PubMed. Studies were included if they assessed cognitive states using physiological signals and applied machine learning (ML) or deep learning (DL) techniques in practical task settings. The review highlights a pivotal shift from shallow ML to DL approaches for analysing physiological signals, driven by DL’s ability to autonomously learn complex patterns in large datasets. By 2023, DL has become the dominant methodology, though traditional ML techniques remain relevant. Additionally, there has been a move from neuroimaging to multimodal physiological modalities, with the decrease in neuroimaging use reflecting a trend towards integrating various physiological signals for more comprehensive insights. Cognitive state recognition is applied across diverse domains such as the automotive, aviation, maritime, and healthcare industries, enhancing performance and safety in high-stakes environments. Electrocardiogram (ECG) is the most utilised modality, with convolutional neural networks (CNNs) being the primary DL approach. The trend in cognitive state recognition research is moving towards integrating ECG signals with CNNs and adopting privacy-preserving methodologies like differential privacy and federated learning, highlighting the potential of cognitive state recognition to enhance performance, safety, and innovation across various real-world applications.

## 1. Introduction

Cognitive states are the various mental conditions that influence how individuals perceive the environment, process thoughts, and make decisions [1]. These states are dynamic, and can be affected by factors such as cognitive workload, which refers to the mental effort required to perform a task; stress levels, which can alter the efficiency of cognitive processing; and external stimuli, which can focus or distract attention. Understanding and assessing cognitive workload is crucial in any situation where matching task demands with human cognitive capacity is essential. This helps ensure that systems are designed to be effective, safe, and user-friendly, and that tasks are appropriate for those who need to perform them.

The formal assessment of cognitive states in task performers is paramount for optimising performance, ensuring safety, and enhancing overall well-being. Such evaluations enable the tailoring of tasks to align with individual mental capacities, thereby improving efficiency and productivity. In environments where safety is critical, monitoring cognitive states helps in averting accidents attributable to factors such as fatigue or cognitive overload. Furthermore, cognitive assessments are integral to identifying specific training needs, facilitating targeted skill development, and the early detection of mental strain, which is essential for timely intervention. They also play a vital role in workload management, preventing burnout by enabling the strategic distribution of tasks. In the realm of human–computer interaction (HCI), understanding cognitive states informs the creation of adaptive interfaces, leading to more intuitive user experiences. Lastly, cognitive state insights in team settings contribute to improved dynamics and cohesion, underpinning collective performance.

The assessment of cognitive states is integral to diverse domains. Research in cognitive science, which often utilises load-induced tasks such as the n-back task, can be applied to human factors and performance optimisation [2,3,4,5,6]. Monitoring cognitive states in surgery can potentially enhance the safety and efficacy of operative procedures [7,8,9]. The ergonomics, automotive, and aerospace industries incorporate cognitive evaluations to optimise the user experience and maintain safety [10,11,12,13]. Occupational safety relies on such assessments to ensure that task performers are cognitively equipped for high-stakes environments, while HCI utilises these insights to refine user interface design. In the military, cognitive readiness is critical for operational effectiveness. Consumer research, artificial intelligence, and machine learning employ cognitive state analysis to predict behaviour and create adaptive systems. The virtual reality and gaming sectors also depend on understanding cognitive states to advance their respective fields, demonstrating the widespread applicability and significance of cognitive state assessments.

The assessment of cognitive states is a multifaceted process that typically involves self-report questionnaires and behavioural observations. The NASA Task Load Index (NASA-TLX) is extensively utilised across various fields to evaluate cognitive workload, and is favoured for its straightforward application and cost-efficiency [14]. Similarly, methods such as the Subjective Workload Assessment Technique (SWAT) and the Workload Profile (WP) are also employed to assess cognitive states in diverse applications [15,16]. However, these self-reported assessment techniques are subjective and only available post-task, thus constraining their deployment in many scenarios where objective, timely, and continuous evaluation is required. To address this issue, neuroimaging and physiological measures are increasingly used to enable cognitive state assessment in more dynamic settings and real-time monitoring. Neuroimaging measures specifically focus on brain function and activity, while physiological measures include monitoring heart rate, skin conductance, and other responses that reflect changes in an individual’s autonomic nervous system. While no single measure directly quantifies cognitive states, a variety of neuroimaging and physiological indicators are employed to provide insights. These include electroencephalography (EEG), which measures electrical brain activity, and functional near-infrared spectroscopy (fNIRS), which assesses brain function. Additionally, measures such as electrocardiography (ECG), eye-tracking, electrodermal activity (EDA), body temperature (TEMP), and electromyography (EMG) reflect changes in an individual’s autonomic nervous system. Importantly, there are other metrics not detailed here, including task performance data and video recordings, that also contribute to the assessment of cognitive states.

Advancements in wireless wearable sensor technology have enabled the measurement of physiological responses without overt behaviour, allowing minimal disruption during task performance and providing a continuous stream of data with negligible delay. As a result, many commercial products have emerged, such as Shimmer [17] and Empatica [18]. These devices are lightweight and wireless, and have been widely adopted in numerous studies for their efficacy in monitoring physiological signals seamlessly during various cognitive tasks [19,20]. In conjunction with these advancements, neuroimaging techniques like EEG and fNIRS, which provide insights into brain activation, are also evolving into more compact designs. Many of these devices, such as those from Artinis [21] and NIRx [22], operate fully wirelessly while maintaining a high sampling rate, enhancing their utility in real-world applications. Muse devices, with the most recent one, Muse S Athena^®^ (InteraXon Inc., Toronto, ON, Canada) [23], were introduced onto the consumer market in March 2025. The Muse S Athena is an advanced version that combines EEG and fNIRS sensors to a compact headband. Indeed, the use of neuroimaging has expanded beyond traditional neuroscience into diverse fields like stress detection in driving [24,25,26], piloting [27,28,29], and surgery [30,31,32]. The integration of EEG and fNIRS into combined systems further contributes to a more comprehensive understanding of brain activity changes [33,34,35]. Moreover, the combination of neuroimaging and physiological modalities has proven highly effective in dynamically assessing cognitive states across various settings, offering a more robust and integrated perspective on individual changes caused by cognitive workload [9,36,37]. Overall, these technological advancements will greatly enhance cognitive state recognition, becoming widespread across diverse fields and improving performance and safety in various activities.

To capitalise on the extensive correlation between various physiological modalities and cognitive states, machine learning approaches have been adopted to discern more intricate associations between cognitive states and physiological fluctuations. Indeed, integrating physiological metrics with machine learning techniques has been successfully applied in numerous studies, yielding precise detection and evaluation of the user’s cognitive workload. Two principal methodologies are predominantly employed in the literature: shallow machine learning (shallow ML) approaches coupled with feature engineering, and deep learning (DL). The former involves manually selecting and crafting features from raw data, then feeding into traditional ML models, like support vector machines (SVM) or decision trees. This process requires domain expertise to identify the most relevant features for accurate cognitive state prediction. On the other hand, DL approaches, such as CNNs and recurrent neural networks (RNNs), automatically extract features through their layered architecture. These methods are particularly adept at handling large volumes of data and can uncover complex patterns that shallow learning techniques might miss. Both approaches offer unique advantages and are chosen based on the specific requirements and constraints of the cognitive state recognition task. In this paper, both shallow ML approaches with feature engineering and DL strategies applied for cognitive state recognition are thoroughly investigated and evaluated. The review systematically selects and analyses the relevant literature, and applies narrative synthesis to interpret and contextualise the findings. This approach offers comprehensive insights into the strengths, limitations, and appropriate application contexts for each method. By bridging a rigorous systematic methodology with interpretative analysis, this review contributes valuable knowledge and practical guidance for researchers and practitioners across a range of cognitive state recognition domains.

### 1.1. Motivation

The recognition of cognitive states is becoming increasingly prevalent in the field of artificial intelligence, with its application extending across a diverse range of domains. This review can significantly contribute to this growing field by providing a comprehensive analysis of current methodologies, applications, and existing limitations.

**Figure 1:**
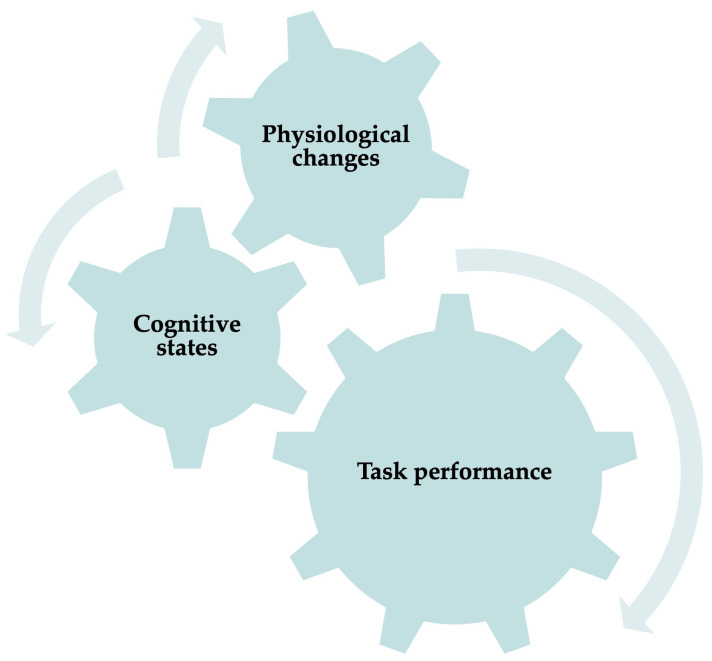
This diagram shows the relationship between physiological changes, cognitive states, and task performance.

The relationship between physiological signal changes, cognitive states, and task performance is intricate and multidimensional. Physiological signals, such as heart rate, skin conductance, and brain activity, can provide objective insights into an individual’s cognitive state. For instance, an increased heart rate and electrodermal activity might indicate heightened stress or cognitive load [38,39]. Figure 1 demonstrates the relationship that closely links physiological responses to cognitive states like attention, stress, and resourcefulness, significantly impacting task performance [40]. By leveraging this relationship, it is possible to ensure task performance through monitoring an individual’s cognitive state. Furthermore, the application of ML techniques facilitates the prompt identification of changes in cognitive states by analysing physiological signals. In practical scenarios, cognitive state recognition plays a pivotal role in modulating task requirements, offering necessary support, or altering the environment to enhance performance and minimise error probabilities. This is especially crucial in high-stakes environments like surgical operations, aviation, or situations demanding critical decision-making.

**Figure 2:**
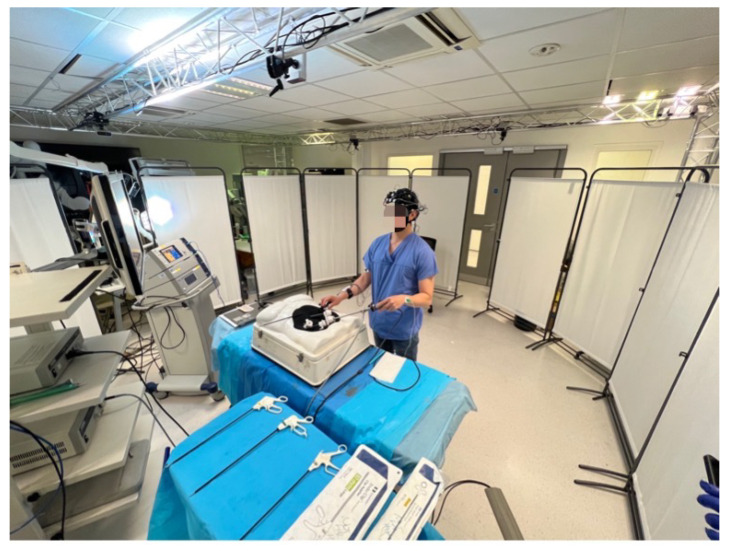
The MAESTRO-simulated OR at St. Mary’s Hospital, London.

Specifically, the motivation for conducting this review also stems from our work on the MAESTRO (Multi-Sensing AI Environment for Surgical Task and Role Optimization) project. The MAESTRO framework, developed by Imperial College London, is designed to monitor physiological changes in the surgical team. To achieve this, a multimodal-sensing environment was built as a prototype at St. Mary’s Hospital, designed to serve as an intelligent simulated operating theatre (shown in Figure 2).

**Figure 3:**
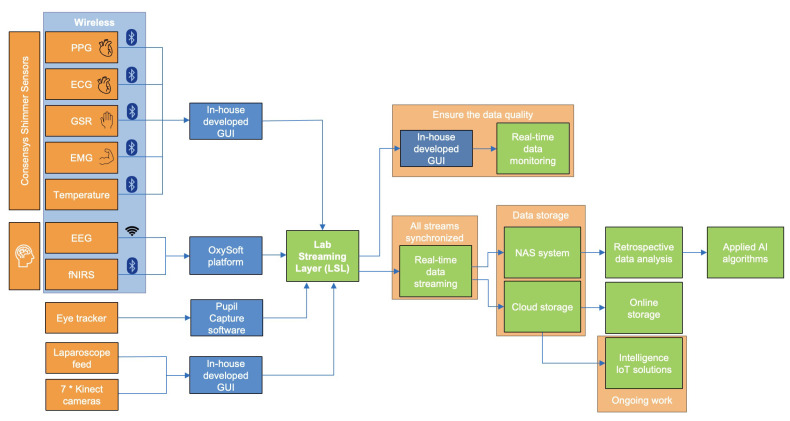
Architecture of the multimodal-sensing platform deployed in the MAESTRO-simulated operating theatre.

The MAESTRO framework employs a range of sensors and cameras, categorised into three main groups. First, general physiological signals are captured using various wearable devices. Pupil metrics are measured with Pupil Core^TM^ (Pupil Labs GmbH, Berlin, Germany) [41] equipment, while Shimmer^TM^ units (Shimmer Research Ltd., Dublin, Ireland) [17] are used to monitor photoplethysmogram (PPG), ECG, GSR, EMG, and body temperature. These physiological signals help assess the surgeon’s cognitive workload, fatigue levels, and gaze/eye movements. Second, neuroimaging signals are recorded using EEG and fNIRS devices. EEG measurements are taken with the Mobita (TMSi, Oldenzaal, The Netherlands) [42] system, while fNIRS data is gathered using the Brite^TM^24 (Artinis Medical Systems B.V., Elst, The Netherlands) [43]. These devices measure electrical activity and blood flow in the surgeon’s brain during procedures, providing direct insights into brain function. Third, video capture is extensively utilised to record various visual aspects of the surgical environment. The eye tracker from Pupil Core^TM^ records the surgeon’s first-person view, and a digitiser card captures the laparoscopic video feed. Additionally, an array of seven Azure Kinect RGB-D cameras (Microsoft Corporation, Redmond, WA, USA) surround the simulated theatre to capture motion and depth information. To synchronise and assemble all sensing modalities mentioned above simultaneously, the Lab Streaming Layer (LSL) [44] platform is employed. The schematic shown in Figure 3 demonstrates the structure of the MAESTRO platform, which is designed to facilitate the holistic sensing of the simulated operating room.

By integrating these diverse data sources, the MAESTRO framework aims to provide a holistic, continuous, and objective assessment of the surgical theatre continuum, alongside a comprehensive view of the cognitive workload and physiological changes experienced by the surgical team for enhancing the safety and efficiency of surgical procedures. Three studies have been conducted to identify surgeons’ cognitive states during surgical tasks such as peg transfer and simulated laparoscopic cholecystectomy. In addition to physiological and neuroimaging data, video was utilised for instrument detection, aiding in the optimisation of the surgical workflow. Diverse modalities combined with DL approaches achieved promising results (details can be found in references [9,45,46,47,48]).

By examining the broader landscape of cognitive state recognition, we aim to align our efforts with emerging trends in recognition approaches and sensing modalities, ultimately enhancing the MAESTRO framework. This review serves to contextualise our research within the wider field, identifying key methodologies and advancements that can inform and improve the development of intelligent operating theatres. Moreover, the flexibility of the MAESTRO framework allows it to be adapted for use in various high-stakes environments, contributing to safer and more efficient practices not only in surgery, but also in other critical fields.

### 1.2. Contribution

**Figure 4:**
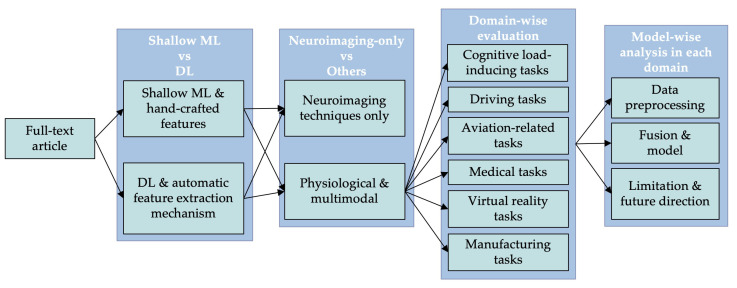
The diagram shows the structure of the review.

This review delves into four comprehensive comparisons or investigations concerning the implementation of ML strategies for recognising cognitive states through the measurement of physiological signals (shown in Figure 4).

The significant contributions offered by this review include:

- **Comparative evaluation:** presenting a comparative analysis of shallow ML versus DL techniques, and neuroimaging versus other physiological modalities, in the realm of cognitive state recognition. It offers valuable insights into the respective strengths and limitations of each approach, thereby informing future research directions and practical applications within the field.
- **Practical application insights:** revealing real-world applications of cognitive state recognition, illustrating its significant impact and potential across various industries such as healthcare, education, automotive, and workplace productivity. Furthermore, the findings provide valuable information and inspiration for future research and development, enhancing the practical utility of cognitive state recognition in multidisciplinary settings.
- **In-depth analysis of Machine Learning strategies:** providing a thorough examination of shallow ML approaches coupled with feature engineering and DL techniques used in cognitive state recognition, highlighting their strengths and limitations. Additionally, the review evaluates how ML techniques have progressed over time, highlighting key advancements, shifts in methodologies, and emerging trends in the context of cognitive state recognition.
- **Integration of Physiological Signals:** this investigation delves into the effective interpretation of physiological signals by DL algorithms for the accurate identification and analysis of various cognitive conditions. It categorises this process based on data preprocessing, modality fusion, and the selection of appropriate DL models, providing a comprehensive understanding of how these elements work in tandem to enhance cognitive state recognition.

## 2. Scope and Methodology of Systematic Literature Review

This review was conducted and reported in compliance with the Preferred Reporting Items for Systematic Reviews and Meta-Analyses (PRISMA) 2020 guidelines [49], ensuring quality, transparency, and reproducibility and enhancing overall robustness and credibility. The completed PRISMA checklist is provided in the Supplementary Materials. However, the review protocol was not registered with PROSPERO, as this review falls under a category (diagnostic/prognostic) that is currently not eligible for registration.

### 2.1. Scope Definition

The objective of this review is to critically evaluate current ML methodologies in cognitive state recognition using physiological signals, focusing on comparing various approaches and exploring the efficacy of DL in interpreting these signals. It aims to guide future research and inform practical applications in the field. Specifically, the following research questions are discussed and explored in this review.

- Q1: What types of ML strategies have been used to classify or predict cognitive states using physiological signals?
- Q2: How has the application of ML in cognitive state recognition evolved, and what are the latest developments in this area?
- Q3: How do different ML strategies, particularly shallow ML versus DL, perform in cognitive state recognition using physiological signals?
- Q4: What types of physiological signals have been used in conjunction with ML to assess cognitive states?
- Q5: What are the strengths and limitations of using neuroimaging modalities versus other physiological modalities in cognitive state recognition?
- Q6: What are the practical applications of cognitive state recognition in fields such as the study investigating the effects of cognitive load-inducing tasks, healthcare, the automotive industry, and workplace productivity?
- Q7: What are the future prospects and potential areas for further research in the development of ML strategies for cognitive state recognition?

### 2.2. Search Strategy and Study Selection

This review conducted a comprehensive analysis of articles published between 2010 and early 2024, focusing on the advancements and developments in the field of cognitive state recognition using physiological signals and ML strategies. The search was performed from a range of reputable databases, including Web of Science, Scopus, PubMed, Engineering Village, and IEEE Xplore, ensuring a comprehensive coverage of literature in the field. The final search across all databases was conducted in February 2024. The search terms, keywords, and Boolean operators listed below were used to ensure a focused and comprehensive literature retrieval.

- **AI-related terms:** “AI” OR “artificial intelligence” OR “deep learning” OR “machine learning” OR “neural network” OR “NN” OR “convolutional neural network” OR “CNN” OR “ConvNet” OR “long short term memory” OR “long short-term memory” OR “LSTM”.
- **Cognitive state-related terms:** “cognitive workload” OR “mental workload” OR “stress level” OR “working memory” OR “cognitive overload” OR “resourcefulness”.
- **Physiological signal-related terms:** “ECG” OR “electrocardiogram” OR “EMG” OR “electromyography” OR “PPG” OR “photoplethysmography” OR “pupil” OR “pupil metric” OR “pupil dilation” OR “blink” OR “eye tracking” OR “GSR” OR “galvanic skin response” OR “fNIRS” OR “functional near-infrared spectroscopy” OR “EEG” OR “electroencephalogram” OR “physiological signal” OR “video” OR “EDA” OR “electrodermal activity” OR “autonomic nervous system".

#### Inclusion and Exclusion Criteria

The articles included in this review were selected based on the specific inclusion and exclusion criteria listed below:

- **Publication date:** articles published from 2010 to early 2024.
- **Topic relevance:** studies focusing on cognitive state recognition using physiological signals and ML strategies. This includes research on cognitive workload (CWL), stress levels, and working memory changes, which are integral aspects of cognitive state assessment and are closely linked to task performance in practical applications. This targeted selection was made to ensure that the review remained focused on real-world CWL evaluation rather than attempting to cover every conceivable cognitive state. Thus, the review excludes studies that address solely mental states—such as emotional changes, imaginative thinking, mind-wandering, and other similar phenomena. Similarly, the review excludes studies that are centred on cognitive training or enhancement methods, such as transcranial direct current stimulation (tDCS) or photobiomodulation (PBM).
- **Physiological signal requirement:** physiological signals are measurable biological responses generated by the human body that reflect activity within the autonomic or central nervous systems, such as cardiac activity (e.g., ECG), neural activity (e.g., EEG), muscle activation (e.g., EMG), or electrodermal responses (e.g., EDA). The signals selected for inclusion in this review represent well-established, objectively quantifiable physiological indicators closely associated with cognitive workload. While other measures, including facial expressions, speech patterns, breathing patterns, and haptics, may also provide insights into cognitive states, they primarily represent behavioural or mixed-modality indicators, and therefore fall outside the specific definition of physiological signals used here. Thus, the studies included in this review were required to utilise at least one physiological signal as a primary data source, with video- or image-based data permitted only as supplementary modalities.
- **Machine learning/deep learning utilisation:** research involving any type of ML or DL approach.
- **Implementation in practical scenarios:** studies must apply cognitive state recognition in practical applications, such as in cognitive load-inducing tasks, healthcare, automotive experiments, workplace productivity, or any other relevant domain.

**Figure 5:**
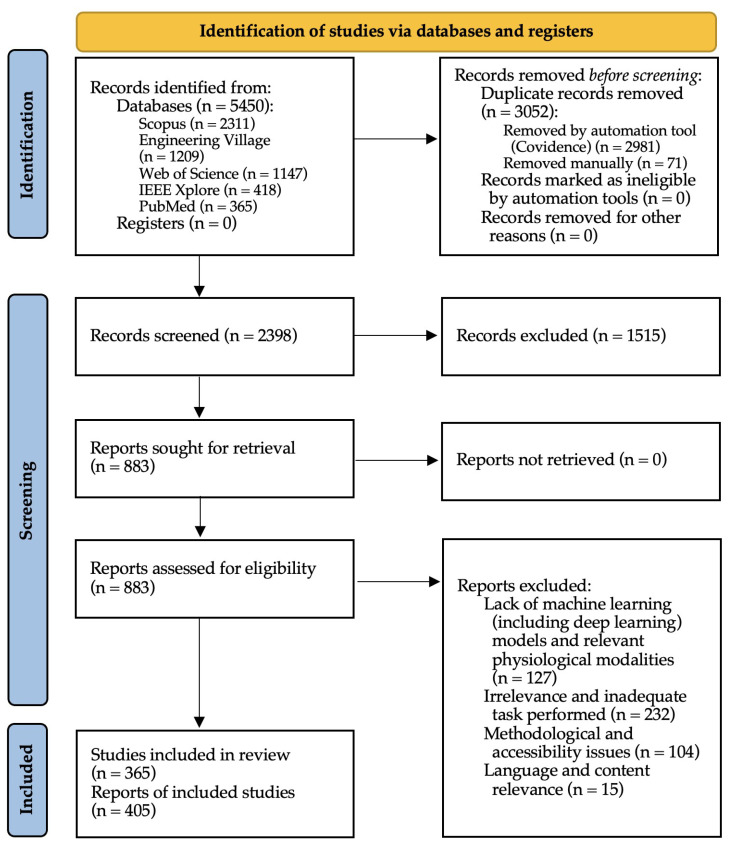
The PRISMA 2020 flow diagram [49] shows the selection process for this review, of 405 relevant articles selected from an initial pool of 2398 studies.

The PRISMA selection process for this review, facilitated by Covidence [50], involved three key stages: identification, screening, and inclusion (shown in Figure 5). Initially, 2398 studies were identified. During the screening stage, studies were evaluated based on their titles and abstracts by two independent reviewers, one PhD student and one PhD researcher, using Covidence. Any disagreements were resolved within the Covidence platform. This stage led to a significant number of exclusions. The final stage involved a thorough assessment of the remaining studies for their full-text eligibility, resulting in 405 studies being included in the review. It is important to note that not all 405 references are cited within the review text, as some were included solely for statistical purposes such as trend analysis.

### 2.3. Data Extraction

The following data items were extracted from each included article through full-text analysis: practical application domain, practical task, classification goal and classes, key physiological features and modalities, deep learning approach used, and classification performance metrics. As this review did not involve intervention outcomes, no formal effect measures (e.g., risk ratios or mean differences) were used. Instead, reported classification metrics, such as accuracy and F1 score, were extracted where available and summarised narratively. These categories were selected to support both trend analysis and structured comparison across studies. Data were extracted locally by one reviewer and verified by a second reviewer to ensure consistency and accuracy. No assumptions were made regarding missing or unclear data. Only information explicitly reported in the articles was extracted. A risk of bias assessment was not conducted, as the focus of this review was on methods and trends rather than on evaluating study quality or outcomes. Similarly, no formal quality appraisal of individual studies was performed using standardised tools, as the substantial methodological heterogeneity across study designs, physiological signals, and analytical approaches made such an assessment inappropriate. The objective of the study was to capture and compare methodological practices rather than to evaluate the strength or efficacy of specific interventions.

### 2.4. Synthesis Approach

Studies were synthesised narratively and grouped according to relevant characteristics, including application domain, cognitive task type, physiological signal modality, and machine learning strategy. This grouping aligned with the predefined research questions and supported comparative insights across categories. The extracted data were prepared manually using spreadsheets and visualised through charts and tables. Classification performance metrics, such as accuracy and F1 score, were extracted when reported in the original studies. However, these metrics were not statistically aggregated or normalised because substantial methodological heterogeneity across studies, including differences in datasets, evaluation protocols and model configurations, prevented meaningful quantitative synthesis. As a result, no formal statistical assessment of heterogeneity or sensitivity analysis was performed. Instead, the synthesis was descriptive in nature and focused on identifying patterns and trends related to modality usage, algorithm selection, and domain-specific applications over time. Moreover, an assessment of reporting bias was not applicable, as no quantitative synthesis or meta-analysis was performed in this review. Certainty of evidence was not assessed, as this review did not involve outcome comparisons or effect estimates.

## 3. A Review of Two Principal Methodologies Employed in Cognitive State Recognition: Shallow Machine Learning Approaches Coupled with Feature Engineering and Deep Learning

As the field of artificial intelligence (AI) has evolved, the topic of cognitive state recognition has gained increasing popularity. This surge in interest is primarily due to the growing understanding of the importance of cognitive states in various applications, such as cognitive workload monitoring, the automobile industry, and healthcare. Within these diverse domains, two principal methodologies have emerged as predominant in the literature: shallow ML approaches coupled with feature engineering, and DL.

In the realm of cognitive state recognition through physiological signals, traditional or shallow ML techniques have established their significance. Central to these methods is the process of feature engineering, where relevant features are meticulously extracted from physiological signals such as ECG, EDA, and EEG. These features often encompass statistical measures and complex time-frequency characteristics, which are pivotal to the model’s performance. Subsequently, these extracted features are processed using common ML algorithms like support vector machines (SVMs) for classification tasks, decision trees for both classification and regression, K-nearest neighbors (KNN) for its simplicity in smaller datasets, and linear or logistic regression for predicting continuous or binary outcomes. The primary advantages of shallow ML methods lie in their simplicity, interpretability, and lower computational demands. However, they often require substantial domain expertise for feature engineering and may not capture the intricate data relationships as effectively as their DL counterparts. This approach, therefore, remains integral in scenarios where interpretability and computational efficiency are paramount.

DL, a more recent development in AI, is increasingly being harnessed for cognitive state recognition through physiological signals, offering a robust solution to decipher complex patterns in large datasets. This approach significantly diminishes the reliance on extensive feature engineering, a notable advantage over traditional ML methods. In the realm of DL, various architectures are employed, each tailored to specific types of physiological data. CNNs are adept at processing time-series data, identifying spatial hierarchies in signals like EEG and ECG. RNNs and Long short-term memory (LSTM) networks excel in analysing sequential data, capturing temporal dependencies crucial for understanding cognitive states. Autoencoders, which are used in unsupervised learning tasks, assist in dimensionality reduction, enhancing data processing efficiency. The primary strength of DL lies in its ability to manage high-dimensional data and unravel complex, non-linear relationships, making it particularly suitable for the intricate nature of physiological signals in cognitive state recognition. However, this approach is not without challenges; it demands substantial data volumes and computational power, and the models’ ’black box’ nature often leads to reduced interpretability. Despite these challenges, DL’s capacity for processing raw data and autonomously learning intricate patterns positions it as an invaluable tool in the evolving landscape of cognitive state recognition. Table 1 shows a comprehensive comparison between shallow machine ML and DL methods, highlighting DL’s superior capabilities in handling large-scale, high-dimensional physiological data and autonomously learning complex patterns, albeit with increased demands for computational resources and reduced model interpretability.

### Development and Evolution of Machine Learning Approaches in Cognitive State Recognition

**Figure 6:**
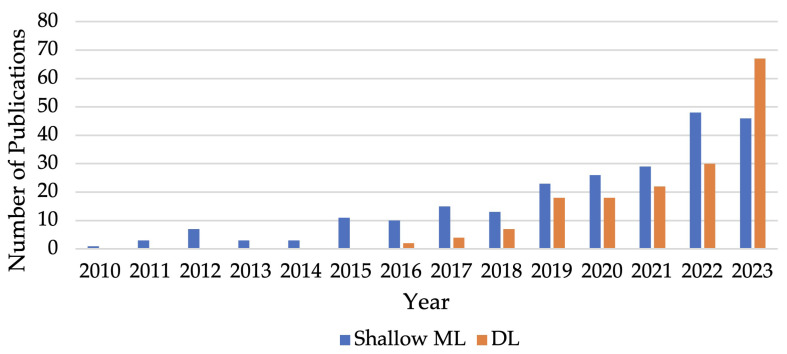
Trend of ML vs. DL publications over recent years.

**Figure 7:**
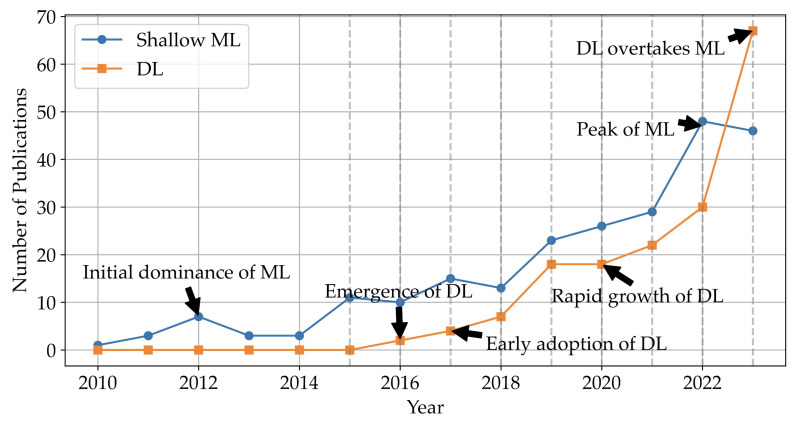
Evolution of shallow ML & DL in cognitive state recognition (2010–2023).

In the domain of cognitive state recognition, the evolution of methodological approaches has been marked by the transition from traditional ML techniques to more sophisticated DL strategies. The data spanning from 2010 to early 2024 reveal a narrative of development that underscores the shifting paradigms within the field (shown in Figure 6). Figure 7 provides a more detailed analysis of the trend, segmenting it into four distinct stages:

- **Early stage (2010–2015):** during the initial years, from 2010 to 2015, shallow ML was the sole approach used in cognitive state recognition, playing the predominant role. This is mostly due to its simplicity and effectiveness for the smaller or structured datasets commonly available at that time. The trend shows a gradual increase in publications, peaking at 11 in 2015. This period emphasises the reliance on domain expertise for handcrafted feature extraction and the application of algorithms that provide a clear understanding of the model’s decision-making process.
- **Emergence of DL (2016–2017):** the landscape began to change in 2016 with the emergence of DL in the literature, marking the beginning of a new era in cognitive state recognition. This period of transition witnesses a slow but steady increase in DL publications, suggesting a growing acknowledgement of its potential to manage larger and more complex datasets without the need for manual feature engineering.
- **Rapid growth of DL (2018–2023):** rapid growth in DL-based cognitive state recognition approaches was observed between 2018 and 2023, with the number of publications increasing from 7 to 67 in 2023. This rapid expansion can be attributed to advancements in computational power, the availability of large datasets, and improvements in neural network architectures, which collectively make DL more accessible and applicable to a broader range of cognitive state recognition problems. Indeed, cross-disciplinary research has increasingly benefited from the capabilities of DL techniques, especially under collaborative frameworks that span multiple disciplines. One of DL’s key advantages is that it does not require advanced knowledge in feature engineering, which reduces the barrier of interdisciplinary collaborations in many specialised fields.
- **Sustained presence and subsequent plateauing of ML (2018–2023):** while DL continues its rapid expansion, shallow ML-based approaches maintain a significant presence, evidenced by a promising development from 2018 to 2022, culminating in a peak of 48 publications in 2022. However, a slight decrease in 2023 indicates a plateau in research activities, which may be suggesting that the field encounters the limitations of shallow learning approaches as DL becomes the dominant modality.
- **Current trends (2023):** in 2023, the data reveals a significant trend as publications on DL (67) outnumber those on shallow ML (46). This signifies a shift in the field of cognitive state recognition, with a growing preference for the multidimensional learning abilities offered by DL models. Furthermore, this takeover reflects a shift in research focus, moving from pursuing more sophisticated feature engineering within ML approaches to the adoption of DL’s capacity for handling larger datasets and a broader spectrum of modalities. This shift is facilitating the evolution of cognitive state recognition toward methodologies that are capable of capturing subtle changes in human cognition across a range of interdisciplinary applications.

In conclusion, the evolution of ML and DL in mental state recognition reflects a vibrant and rapidly evolving field; while DL has become increasingly prominent due to its robustness in handling complex data, ML maintains a significant presence. The future may see more hybrid approaches, leveraging the strengths of both ML and DL to push the boundaries of what is possible in mental state recognition, leading to more sophisticated applications in cognitive studies, healthcare, and more interdisciplinary fields.

## 4. Role of Neuroimaging Signals in Assessing Cognitive States

The role of neuroimaging signals, particularly EEG and fNIRS, in assessing cognitive states is pivotal due to their direct measurement of brain activity, offering insights into various cognitive processes. Leveraging the ability of ML approaches to analyse complex patterns in data, EEG and fNIRS have become increasingly valuable in cognitive state recognition. These technologies enable the extraction of meaningful insights from the intricate neural signals captured by EEG and fNIRS, enhancing our understanding of cognitive processes.

**Figure 8:**
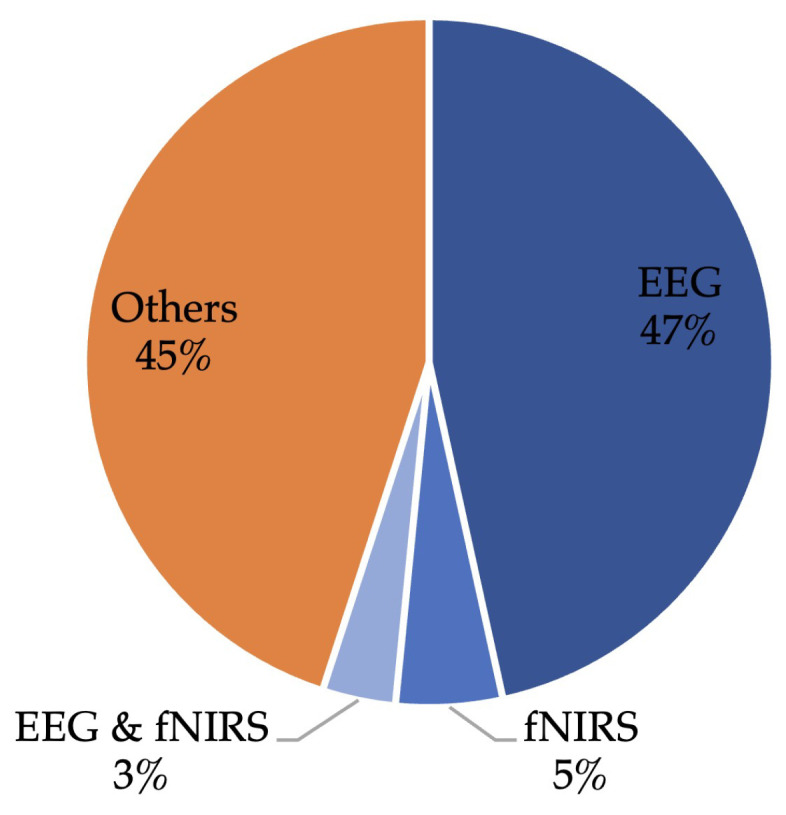
Proportional distribution of cognitive state recognition research by neuroimaging modality.

In this comprehensive review, a significant portion of the analysed literature, specifically 218 out of the 405 articles, exclusively focuses on the utilisation of neuroimaging signals for cognitive state recognition (as shown in Figure 8). These studies are categorised based on their primary neuroimaging modality, encompassing those employing solely EEG, solely fNIRS, and a combination of EEG and fNIRS. The “Others” category in Figure 8 includes studies that incorporate a broader range of physiological signals beyond EEG and fNIRS, such as ECG, EMG, PPG, pupil metrics, and other peripheral measures. Some of these studies include EEG or fNIRS as part of a broader set of physiological signals; however, their primary focus is not on neuroimaging. Rather, they adopt a multimodal framework in which all modalities are considered collectively to support cognitive state recognition.

- **Solely EEG in cognitive state recognition:** EEG is highly effective in cognitive state recognition due to its ability to capture rapid, millisecond-level fluctuations in brain activity. This high temporal resolution is essential for analysing dynamic cognitive processes such as attention shifts, mental workload, and emotional responses. EEG signals are typically analysed through features like power spectral density, coherence, and event-related potentials (ERPs) [51]. These features provide insights into various frequency bands (alpha, beta, gamma, delta, theta) associated with different cognitive states. ML and DL techniques, such as SVMs and CNNs, are employed to classify these states based on EEG features. However, EEG’s susceptibility to artefacts and its limited spatial resolution, which restricts the ability to pinpoint the exact location of brain activity, poses challenges in data interpretation.
- **Solely fNIRS in cognitive state recognition:** the fNIRS signal provides a window into cerebral blood flow and oxygenation, correlating with neural activity during cognitive tasks. It offers spatial insights into brain function, particularly in areas like working memory and executive functions. In fNIRS, the concentration changes of oxygenated and deoxygenated haemoglobin are key features used in cognitive state analysis. The application of ML and DL to fNIRS data has opened new avenues for cognitive state classification. Despite its advantages, including lower susceptibility to electrical artifacts compared to EEG, fNIRS faces challenges like lower temporal resolution and sensitivity to extracerebral factors.
- **Combining EEG and fNIRS:** the integration of EEG and fNIRS data marks a significant advancement in cognitive state recognition, merging EEG’s temporal accuracy with fNIRS’s spatial resolution. This multimodal approach allows for a comprehensive analysis, where EEG’s sensitivity to rapid cognitive shifts complements fNIRS’s ability to localise brain activity during sustained tasks. Developing multimodal ML/DL models that effectively combine features from both EEG and fNIRS is at the forefront of current research. This synergy enhances the accuracy in identifying complex cognitive states, although it introduces challenges in data synchronisation, integration, and increased computational demands. A comparative summary of these three neuroimaging modalities, including their advantages, limitations, and application contexts in cognitive state recognition, is provided in Table 2.

Apart from cognitive state recognition using solely neuroimaging modalities like EEG and fNIRS, other physiological modalities such as ECG, EMG, and pupil metrics also play a significant role in this field. These modalities offer different perspectives and insights into an individual’s cognitive state.

**Table 2:** Detailed comparison of EEG, fNIRS, and EEG and fNIRS in cognitive state recognition.

Modality	Number of Studies	Pros	Cons	Case Studies and Methodological Considerations
EEG [13,24,25,26,27,28,30,31,32,52,53,54,55,56,57,58,59,60,61,62,63,64,65,66,67,68,69,70,71,72,73,74,75,76,77,78,79,80,81,82,83,84,85,86,87,88,89,90,91,92,93,94,95,96,97,98,99,100,101,102,103,104,105,106,107,108,109,110,111,112,113,114,115,116,117,118,119,120,121,122,123,124,125,126,127,128,129,130,131,132,133,134,135,136,137,138,139,140,141,142,143,144,145,146,147,148,149,150,151,152,153,154,155,156,157,158,159,160,161,162,163,164,165,166,167,168,169,170,171,172,173,174,175,176,177,178,179,180,181,182,183,184,185,186,187,188,189,190,191,192,193,194,195,196,197,198,199,200,201,202,203,204,205,206,207,208,209,210,211,212,213,214,215,216,217,218,219,220,221,222,223,224,225]	184 Period: 2010–2023	- Exceptional temporal resolution, ideal for analysing rapid cognitive dynamics. - Versatile in assessing a range of cognitive states. - Effective in studying attention, emotional responses, and sleep stages.	- Limited spatial resolution, not precise in pinpointing brain activity locations. - Susceptible to noise and artifacts, requires sophisticated filtering. - Interpretation can be complex due to signal variability.	The popularity of EEG (184 studies) underscores its utility in capturing fast-changing brain dynamics, despite spatial resolution and noise challenges.
fNIRS [29,226,227,228,229,230,231,232,233,234,235,236,237,238,239,240,241,242,243,244,245]	21 Period: 2017–2023	- Offers spatial insights into brain activity, especially in localised regions. - Less affected by electrical artifacts compared to EEG. - Useful in studying cognitive functions like working memory and executive functions.	- Lower temporal resolution than EEG, slower in detecting changes. - Can be sensitive to scalp blood flow, affecting signal purity. - Requires careful interpretation to differentiate cerebral from extracerebral signals.	The lower number of fNIRS studies (21) reflects its limitations in temporal resolution, but its spatial accuracy provides valuable insights into brain function.
EEG & fNIRS [33,34,35,246,247,248,249,250,251,252,253,254,255]	13 Period: 2017–2023	- Combines EEG’s temporal accuracy with fNIRS’s spatial resolution. - Provides a comprehensive analysis of brain activity. - Enhances accuracy in cognitive state recognition, covering both fast and localised brain changes.	- Integrating data from both modalities can be complex. - Increased computational demands for processing and analysis. - Challenges in synchronising and interpreting combined data.	The combination approach, used in 13 studies, is less common due to integration complexities but offers a promising comprehensive view of cognitive states.

### Emerging Trends in Modalities for Cognitive State Recognition

**Figure 9:**
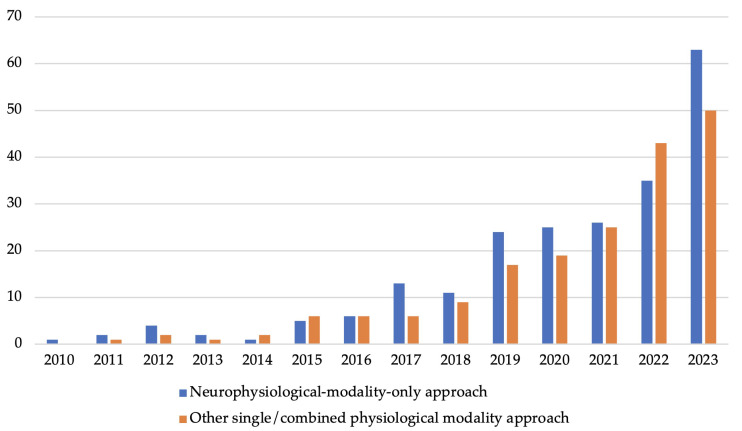
Trends in neuroimaging and combined physiological modalities: 2010–2023.

In cognitive state recognition, there has been a notable shift in the modalities used over the past decade. The focus has moved beyond traditional neuroimaging to include diverse physiological signals. Figure 9 illustrates this trend across studies from 2010 to 2023, highlighting the growing integration of multimodal data for richer cognitive analysis. The category ‘Neurophysiological-modality-only approach’ refers to studies that exclusively use neuroimaging signals, such as EEG and fNIRS, to recognise cognitive states. In contrast, the ‘Other single/combined physiological modality approach’ category includes studies that use either a single physiological signal such as ECG, EMG, or PPG, or combine multiple physiological signals (as a multimodal input), including both neuroimaging and peripheral measures.

- **Initial stage (2010–2015):** during the initial stage, from 2010 to 2014, research was primarily focused on neuroimaging modalities alone, specifically using EEG. This period marked the advent of utilising direct brain activity measurements to interpret cognitive states. Techniques like Fourier transform were commonly employed for feature extraction in the frequency domain, laying the groundwork for the application of shallow ML algorithms in the analysis and interpretation of these signals [136,172]. In 2011, cognitive state recognition research began to diversify with the emergence of studies incorporating other single or combined physiological modalities such as ECG, EMG, and GSR. These signals were processed similarly to neuroimaging modalities, utilising shallow ML on hand-crafted features [256,257,258]. This trend suggested an exploratory phase, indicating early exploration in the adoption of multimodal approaches.
- **Growth and diversification (2016–2023):** during this stage, both approaches exhibit a steady increase in studies. This consistent growth indicates a growing interest in diversifying cognitive state recognition methodologies. As the field progressed from 2016 onwards, there was a marked increase in the adoption of the other single/combined physiological modality approach, which began to gain significant traction by 2017, evidenced by a swift escalation in publication numbers. In 2022, publications focusing on physiological modality approaches outnumbered those dedicated solely to neuroimaging modalities, signalling a shift in research direction within the field. However, in 2023, the neuroimaging-modality-only approach regained its lead in the number of publications. In 2023, 40 out of 63 studies focusing solely on neuroimaging modalities utilised DL, nearly doubling the count from 2022, where only 21 out of 55 studies employed DL techniques. Similarly, in 2023, 27 out of 50 studies involving physiological modalities utilised DL, a significant increase from 2022, where only 9 out of 43 studies employed DL techniques. Both demonstrate an increasing trend towards using DL to interpret data from different modalities, highlighting DL’s growing importance in cognitive state recognition research.
- **Current trend (2023):** to summarise, the comparative trend analysis from 2015 to 2023 underscores a significant shift from primarily using solely neuroimaging modalities with shallow ML to adopting multimodal physiological modalities enhanced by DL.

The trend analysis suggests that while neuroimaging modalities provided a strong foundation for cognitive state recognition, the field is evolving towards a more integrated approach that combines various physiological signals. Indeed, between 2011 and 2020, 13 out of 67 (19.4%) studies in cognitive state recognition utilised neuroimaging modalities. Comparatively, in 2023, only 8 out of 50 (16%) multimodal approaches included neuroimaging. This demonstrates a decreasing reliance on neuroimaging as part of multimodal studies, reflecting the field’s shift towards more diverse physiological signal integration. This shift is driven by the desire to overcome the inherent limitations of relying on neuroimaging signals, such as the complexity of setups, the expertise required for interpretation, and the potential for more comprehensive assessments using multimodal data. Notably, Figure 9 shows a marked increase in the use of other single or combined physiological modalities beginning in 2018. This upward trend aligns with the early adoption of deep learning observed in Figure 7, suggesting a close relationship between the advancement of DL techniques and the growing feasibility of interpreting peripheral physiological signals. DL methods, particularly CNNs and RNNs architectures, have enabled researchers to extract complex, meaningful features from raw signals such as ECG, EMG, and PPG without the need for extensive handcrafted feature engineering. This has significantly boosted the capability to integrate and interpret multimodal physiological data, contributing to the broader shift away from neuroimaging-only approaches. The movement towards the multimodal physiological modality approach is not just a current trend, but is also likely to continue into the future, shaping the trajectory of cognitive state recognition research. Nevertheless, in some scenarios, combining physiological and neuroimaging modalities can still potentially provide a more holistic view of cognitive states.

## 5. Revealing Real-World Applications of Cognitive State Recognition

**Figure 10:**
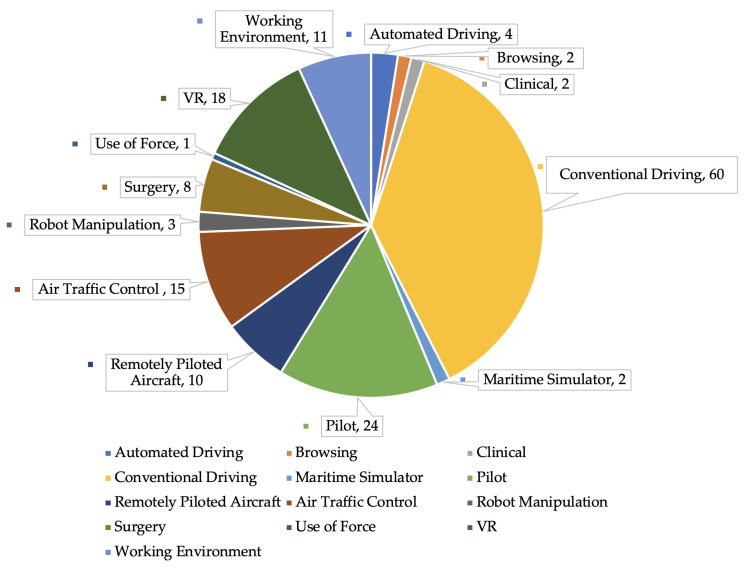
Distribution of research studies by application in cognitive state recognition across various domains.

This systematic review analyses research across multiple domains to identify and understand real-world applications involving cognitive state recognition (shown in Figure 10). Cognitive load-inducing tasks lead, with 245 studies, emphasising their central role in cognitive state recognition.

With a total of 64 studies, driving tasks, including conventional (60) and automated (4) tasks, rank as the second most researched area. This highlights the automotive sector’s commitment to understanding cognitive stress, intending to enhance safety and drive technological innovation. Research in aviation-related tasks underscores the importance of cognitive state recognition in enhancing aerial system performance. This review identifies a total of 49 relevant studies, comprising 24 on conventional piloting tasks (both simulated and real-world flight), 10 on remotely piloted aircraft operations (such as drone and UAV control), and 15 on air traffic control. In this review, ‘aviation-related tasks’ is used as a general category encompassing these three subdomains. Collectively, this body of research is crucial for improving pilot performance, reducing operational errors, supporting remote flight technologies, and enhancing situational awareness and decision-making in air traffic management. Applying these insights to maritime simulators and use-of-force scenarios emphasises the broad applicability of cognitive state recognition in complex and high-stakes environments, highlighting its role in ensuring proficiency and safety across various domains. Virtual reality (VR), featured in 18 studies, provides an immersive environment for cognitive state recognition. Its ability to simulate complex, real-world scenarios in a controlled setting enhances the understanding of cognitive load and decision-making, making VR a valuable tool in psychology, education, and training. Studies on robot manipulation (3), browsing (2), and the working environment (11) explore cognitive state recognition in production and workplace settings. Research in robot manipulation enhances human–robot interaction for industrial efficiency, while browsing studies focus on optimising information retrieval interfaces. Work environment research aims to boost productivity and safety by managing cognitive load among employees, highlighting the role of cognitive recognition in improving workplace dynamics and ergonomics. Research in clinical settings (two studies) and surgery (eight studies) emphasises the importance of understanding healthcare professionals’ cognitive states to enhance communication and patient safety. In this review, both surgical and clinical tasks are grouped under the broader category of medical tasks. Surgical tasks refer to activities conducted in simulated or real operative environments, while clinical tasks include diagnostic and monitoring procedures, such as ultrasound scanning. Recognising cognitive stress in surgeons can prompt timely support, reducing errors and improving outcomes. Similarly, in clinical environments, a better awareness of staff’s cognitive loads can lead to more effective patient care practices, helping prevent misdiagnoses and treatment errors, thus ensuring a safer healthcare system.

The systematic review underscores the pivotal role of cognitive state recognition across a spectrum of domains, demonstrating its essential utility in enhancing performance, safety, and innovation in diverse professional and everyday settings. From the dominant research focus on cognitive load tasks to specialised applications in driving, aviation, virtual reality, and healthcare, the findings reveal a comprehensive approach to understanding and optimising human cognitive capacities. This cross-disciplinary interest highlights the potential of cognitive state recognition to fundamentally improve operational efficiency, reduce error rates, and elevate safety standards, thereby making a significant impact on technological development and quality of life.

Given the unique focus of this review on studies that utilise multimodal physiological modalities and DL models, a more detailed evaluation was conducted on a study-by-study basis. This specialised approach enables a thorough assessment of how integrating various physiological signals with advanced computational techniques enhances the accuracy and applicability of cognitive state recognition. This detailed analysis specifically addresses the effectiveness of these integrative methods in capturing complex cognitive dynamics across different real-world settings.

### 5.1. Multimodal Physiological Modalities and Deep Learning in Cognitive State Recognition Across Real-World Applications

**Figure 11:**
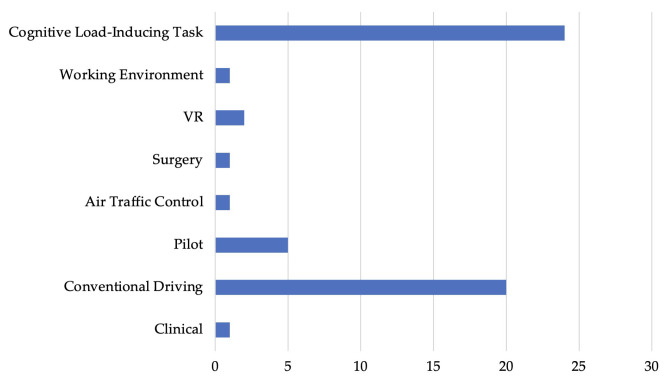
Distribution of studies employing multimodal physiological modalities and DL across various cognitive state recognition tasks.

In this subsection, we explored 55 out of the 405 studies that employ multimodal physiological modalities with DL models, marking a trending approach in cognitive state recognition. These selected studies represent the cutting edge in leveraging diverse physiological data with advanced algorithms to improve the accuracy and application of cognitive assessments across multiple fields. As shown in Figure 11, the data reflects a targeted selection of research across various domains. With 24 studies in cognitive load-inducing tasks and 20 in conventional driving leading the distribution, the analysis is designed to unravel the complexities of these methods, examining their effectiveness in precise cognitive state identification and their capacity to improve practices within specific domains.

#### 5.1.1. Cognitive Load-Inducing Task

This subsection examines 24 of the 55 studies (shown in Table 3) focused on cognitive load-inducing tasks, which are activities designed to create measurable stress on cognitive faculties, serving as a foundation for developing cognitive state recognition models. These tasks range from simulated environments to problem-solving activities, effectively mirroring real-world cognitive challenges. Problem-solving activities such as solving verbal logic puzzles, mental arithmetic tasks, and the Stroop task [259] effectively engage cognitive faculties, offering a reliable measure of cognitive load and stress in controlled settings. Standardised tasks like the N-back task [260] are also utilised, providing a quantitative framework to assess memory and attention across various cognitive load conditions. Additionally, the use of public datasets, notably WESAD [261] and MAUS [262], provides consistent data for a better evaluation of DL models, enhancing the reliability and comparability of research findings across studies.

A wide range of physiological modalities are employed to capture changes in cognitive states. ECG is the most frequently used modality, appearing in 12 studies; it primarily monitors heart function, with both raw time-series data and heart rate variability (HRV) frequently utilised to assess autonomic nervous system activity. This is followed by EDA and GSR, both measuring skin conductance in a combined total of 13 studies. PPG and RESP, each in seven studies, assess blood flow and breathing patterns, respectively, highlighting cardiovascular and respiratory responses to cognitive load. TEMP and ACC are used in six studies each, tracking body temperature fluctuations and physical movements. EEG appears in four studies, essential for measuring brain activity and neural responses, while EMG, also in four studies, evaluates muscle activity. Pupilometry is utilised in five studies to monitor changes in cognitive load and stress through pupil diameter and blink rates. Notably, one study by Youngjun Cho [263] obtains blink time-series data from RGB camera images rather than traditional eye-tracking glasses, showcasing the adaptability of pupilometry in different experimental setups. Less common but significant, BVP appears in two studies and fNIRS and facial features are each used in one study, offering insights into cardiovascular dynamics and emotional states, respectively. This extensive use of diverse physiological modalities ensures a comprehensive and nuanced understanding of cognitive states, allowing researchers to adapt devices as needed in various scenarios.

DL techniques range from traditional DNN, CNN, and RNN to more advanced models like CNN-LSTM hybrids and transformers, which are used to enhance data analysis by integrating both spatial and sequential learning capabilities. CNNs, utilised in nine studies, are the most frequently applied technique due to their superior spatial capabilities for feature extraction, effectively bridging the gap between raw physiological data and interpretable insights. When used solely as feature extraction mechanisms, CNNs are seamlessly integrated with fully connected layers, LSTM networks, and even shallow ML methods, further enhancing their utility and making them a trending approach in the field of cognitive state recognition. CNNs can also employ innovative methods like Gramian Angular Field image encoding to transform time-series data into formats more amenable to CNN processing [4]. The CNN-LSTM hybrid model, featured in six studies including four from 2023, merges CNNs’ spatial feature extraction with LSTMs’ temporal analysis capabilities. This combination is particularly effective for handling complex physiological data that exhibits both spatial and temporal characteristics, such as ECG signals, which are involved in five out of the six studies. This model ranks as the second most utilised approach, reflecting its growing prominence and widespread adoption for its robust capacity to analyse dynamic cognitive states. The adoption of attention mechanisms and transformers collectively enhances the analysis of physiological signals in cognitive state recognition [264,265,266]. Attention mechanisms prioritise relevant data features, significantly improving accuracy in assessments of stress and workload. Meanwhile, transformers, adapted from natural language processing, are promising in analysing time-series data due to their efficiency in capturing long-range dependencies. This makes them ideal for continuous physiological monitoring, where understanding temporal patterns is crucial. Together, these technologies optimise the focus and scope of data analysis, ensuring precise and comprehensive evaluations of cognitive states. Furthermore, the integration of privacy-preserving frameworks like differential privacy (DP) and federated learning (FL) plays a crucial role in the analysis of sensitive physiological data by safeguarding individual privacy [265,267,268]. DP introduces randomness into the dataset to prevent the identification of individual data points, while FL allows models to be trained directly on devices, without needing to share the data itself. These frameworks ensure that personal health information is protected, which not only complies with privacy laws, but also expands the potential for conducting research in environments where data sharing is restricted.

**Table 3:** Table of studies on cognitive load-inducing tasks using multimodal physiological modalities and deep learning (2010–2023).

Authors	Practical Application	Practical Task	Classification Goal & Classes	Key Features & Modalities	Deep Learning Approach	Classification Performance
Dolmans et al. (2020) [36]	Mental workload classification in brain–computer interface	Solving verbal logic puzzles	Classification of perceived mental workload into seven levels	fNIRS, GSR, PPG, and eye-tracking	Intermediate fusion multimodal DNN	Seven-class: 98.5% accuracy
Mozafari et al. (2020) [269]	Stress detection in IoT settings	Mental Arousal Level Recognition Competition database	Five cognitive stress levels (one rest and four stress levels)	GSR, PPG, and RESP	Shallow ML and CNN with PCA	CNN has then lowest accuracy compared to SLR, SVM, and LDA
Hu et al. (2021) [270]	Mental fatigue monitoring	Modified N-back task	Real-time estimation of mental fatigue levels	ECG, RESP, and pupil diameter	LSTM network	Average RMSE of 0.13, Pearson’s R of 0.70 under 20-fold cross-validation
Rashid et al. (2021) [271]	Stress recognition for wristwatches	WESAD dataset	Three-class and two-class classification (stress levels)	Blood volume pulse (BVP) collected from wrist-based PPG	Hybrid CNN (H-CNN)	3-class: 75.21% accuracy and macro F1 64.15%; 2-class: 88.56% accuracy and macro F1 86.18% under LOSO calidation
Youngjun Cho (2021) [263]	Mental workload assessment	Mental arithmetic task	Assessing task difficulty into two levels	Spontaneous eye-blink time-series via RGB camera	Multi-dimensional LSTM network	Over 70% mean accuracies in all labelling strategies
Ghosh et al. (2022) [4]	Stress detection from physiological sensors	WESAD dataset	Stress classification into three levels	ACC, ECG, TEMP, RESP, EDA, and EMG	CNN with Gramian Angular Field image encoding	Three-class: 94.77% accuracy, 0.95 precision, 0.95 recall, and 0.95 F1 score
Tanwar et al. (2022) [272]	Stress detection from physiological sensors	WESAD dataset	Three-class stress classification (baseline, stress, amusement)	ECG, EMG, TEMP, EDA, and RESP	CNN-LSTM	Three-class: 90.20% accuracy
Seo et al. (2022) [273]	Work-related stress detection	Stroop task	Two-level and three-level stress classification	ECG, RESP, and facial features	Deep neural network with feature-level and decision-level fusion	Two-level: 73.3% accuracy, AUC of 0.822, F1 score of 0.700; three-level: 54.4% accuracy, AUC of 0.727, F1 score of 0.508
Shermadurai et al. (2023) [274]	Stress classification from physiological sensors	DEAP and WESAD datasets	Three-class stress classification (low, medium, high stress)	EEG and acceleration (ACC)	CNN (as feature extraction mechanism) with data fusion	Three-class: 82.85%
Mirzaeian et al. (2023) [275]	Cognitive workload estimation	Arithmetic task with different workload levels	Discrimination of three workload levels	EDA using smooth pseudo-Wigner–Ville distribution (SPWVD) and gray-level co-occurrence matrix (GLCM)	Cascade-forward neural network (CFNN), RNN	Three-level: 97.71% average accuracy
Kuttala et al. (2023) [276]	Stress detection using physiological signals	N-back task (MAUS dataset)	Binary classification of stress level	EDA and ECG	Hierarchical CNN with multimodal feature fusion using multimodal transfer module (MMTM)	Two-class classification achieved 88.7% accuracy, 0.88 F1 score, and 0.87 AUC.
Lange et al. (2023) [265]	Privacy-preserving stress detection	WESAD dataset	Binary stress detection (stress vs. non-stress)	BVP, EDA, TEMP, and ACC	Time-series classification transformer (TSCT) with differential privacy (DP)	Accuracy: 91.89% (non-private baseline), 78.16% (ϵ = 1), F1-score: 91.61% (non-private baseline), 71.26% (ϵ = 1)
Zhang et al. (2023) [277]	Cognitive load recognition	Arithmetic computation tasks	Binary classification of cognitive load levels (relaxed vs. intense)	Finger-clip PPG sensor data	LSTM model	92.3% binary classification accuracy
Sarker Bipro et al. (2023) [278]	Mental stress detection	N-back tasks in Mental Arithmetic Stress Dataset (MAUS)	Binary classification of stress levels	ECG	CNN-LSTM model	Accuracy: 75%, sensitivity: 70.37%, specificity: 84.62%, precision: 90.48%, F1-score: 79.17%
Guo et al. (2023) [279]	Mental stress detection	Mental arithmetic tests	Four-class mental stress classification (rest, low, medium, high)	R-R interval, GSR, and RESP	CNN-based models	Identification accuracy varies with stress levels
Singh et al. (2023) [280]	Stress classification from physiological sensors	WESAD dataset	Three-class stress classification	EDA, ECG, RESP, EMG, and TEMP	Hybrid CNN-LSTM model	90.45% accuracy, F1-score 90.28%
Melvillo et al. (2023) [281]	Stress detection using ECG signals	WESAD dataset	Binary classification of stress states	ECG	Combined CNN and LSTM model	Achieved highest accuracy of 97.07% using HRV data in both time and frequency domains
Chen et al. (2023) [282]	Stress analysis in university students	University students playing Sudoku under different conditions	Stress level classification under three scenarios (relaxed, medium, high stress)	ECG, PPG, and EEG	Enhanced models like LRCN and self-supervised CNN	High accuracy in all scenarios, with up to 98.78% accuracy and F1-scores up to 96.67%
Saleh et al. (2023) [37]	Mental workload recognition	N-back task with three difficulty levels	Classification of increasing, stable, or decreasing mental workload	EEG, EDA, PPG, and eye-tracking	CNN with novel merging layer	Superior accuracy compared to classical CNN, BiLSTM, and transformer networks across multiple data modalities
Feng et al. (2023) [20]	Affect and stress detection	WESAD dataset	Binary classification of street level (stress vs. non-stress)	ECG, EMG, EDA, TEMP, and ACC	Feature-level fusion of LSTM and 1DCNN for spatial and temporal feature extraction	Accuracy: 94.9%, F1-score: 94.98%
Benouis et al. (2023) [267]	Privacy-preserving stress recognition	WESAD dataset	Binary classification of street level (stress vs. non-stress)	PPG, EDA, and ACC	Multi-task learning combining federated learning and differential privacy	Achieved accuracy of 90%, identity re-identification limited to 47%
Soni et al. (2023) [266]	Stress detection	WESAD dataset	Binary classification of street level (amused vs. stressed)	ECG, EDA, TEMP, and ACC	Multi-layered DL approach using AutoEncoders, LSTM, and transformers	Achieved a stress detection rate of 98%
P. Mukherjee and A. Halder Roy (2023) [264]	Stress recognition	Solving mathematical problems of varying complexity	Classification of four stress levels (no stress, low stress, medium stress, high stress)	EEG and PPG	Attention mechanism-based CNN-LSTM model	Average accuracy of 97.86%
Fenoglio et al. (2023) [268]	Privacy-aware cognitive workload estimation	Four distinct activities (COLET dataset)	Two-level cognitive workload (low, high)	Eye-tracking	Federated learning (FL), CNN	Average accuracy on LOSO validation: 95.40%

#### 5.1.2. Conventional Driving

This subsection examines 20 of the 55 studies that focus on conventional driving tasks, each leveraging various deep learning techniques to assess drivers’ cognitive states such as stress, workload, and engagement. The tasks involved in the studies can be divided into two categories: simulated driving scenarios, which provide a controlled environment for detailed cognitive assessments, and real-world driving tasks, which capture more dynamic and unpredictable data reflective of actual driving conditions. Furthermore, in some simulated driving tasks, a standardised approach such as the n-back task is used as the secondary task to add quantifiable cognitive load to the driver. This method helps precisely measure the impact of cognitive demands on driving performance under controlled conditions.

A variety of modalities are utilised to measure physiological and environmental factors influencing driver behaviour. ECG, the most prevalent and featured in 14 studies, provides crucial insights into heart activity, where raw signal data, heartbeats, and HRV are used by DL models to assess stress and workload levels in drivers. EDA and GSR, which measure skin conductance to detect emotional and cognitive arousal, are the second most utilised modalities, featured together in 10 studies. Pupilometry, utilised in five studies, tracks changes in pupil size and gaze movement. RESP, featured in four studies, monitors breathing patterns. EEG, used in three studies, measures brainwave activity to assess cognitive functions and mental states exclusively in simulated driving settings. TEMP, which is only featured in one study, tracks body temperature fluctuations. Additionally, vehicle data, road scene video, and head pose, each featured in 2 studies, capture crucial environmental and behavioural aspects of driving. Vehicle data, such as vehicle kinematics, tracks the car’s operational metrics, providing insights into how the vehicle manoeuvres and responds under various conditions. Road scene video offers contextual visibility of the driving environment, helping to analyse how external factors influence driver decisions. Meanwhile, the head pose assesses the driver’s orientation and attention, critical for evaluating driver alertness and focus. Together, these modalities provide a comprehensive view of the external influences on driver performance.

The DL approaches utilised in the conventional driving tasks, show similarities to those in cognitive load-inducing tasks, with CNNs featuring prominently in 11 studies, making them the most utilised technique. This prevalence highlights CNNs’ effectiveness in extracting spatial features from various types of data, which is crucial for analysing visual inputs like road scene videos [11,283]. Additionally, CNNs are adept at processing transformed physiological signals such as ECG, EMG, and EDA [284,285,286,287]. Techniques like spectrograms, Continuous Wavelet Transforms, and scalograms convert these signals into image formats, enabling CNNs to analyse these complex data types effectively, thus providing comprehensive insights into drivers’ physiological states during different driving conditions. Furthermore, the use of pre-trained CNN models such as ResNet50, GoogLeNet, and DenseNet-201 further enhances their utility, leveraging their pre-existing sophisticated feature-extraction capabilities to improve their accuracy and reduce their training time [285,286]. The adaptability of CNNs to handle both physiological and environmental data effectively makes them particularly suitable for the dynamic and visually rich context of driving tasks. Whereas, CNN-LSTM models are only featured in two studies, and transformers appear in just one study, suggesting their less frequent utilisation compared to CNNs in conventional driving tasks.

Table 4 lists all 20 conventional driving studies analysed above. These studies highlight the critical intersection of automotive safety and cognitive state recognition, illustrating their substantial contributions to the development of smarter, safer automotive technologies. By effectively analysing and adapting to drivers’ mental states, these advancements can predict and mitigate potential risks, thereby reducing accidents and enhancing the overall driving experience.

**Table 4:** Table of studies on conventional driving tasks using multimodal physiological modalities and deep learning (2010–2023).

Authors	Practical Application	Practical Task	Classification Goal and Classes	Key Features and Modalities	Deep Learning Approach	Classification Performance
Wang et al. (2018) [288]	Automotive safety	Simulated driving	Cognitive workload demand assessment (low, high)	Eye-gaze patterns	m-HyperLSTM	Precision: 83.9%, recall: 87.8%
Aghajarian et al. (2019) [289]	Automotive safety	Simulated driving	Hazardous driver states (multiple classes: drowsiness, high traffic, adverse weather, cell phone usage)	ECG, RESP, TEMP, GSR, and vehicle kinematics	PCA and ANNs	Accuracy: 75.9% (cell phone usage), 82.7% (alert vs. drowsy), 81.5% (highway vs. town), 71.1% (snowy vs. clear)
Rastgoo et al. (2019) [11]	Driver stress detection	Simulated driving in a driving simulator	Driver stress levels (low, medium, high)	ECG, vehicle data, and environmental conditions	CNN-LSTM network	Average accuracy: 92.8%, sensitivity: 94.13%, specificity: 97.37%, precision: 95.00%
Xie et al. (2019) [290]	Automotive	Real-world driving	Driver mental workload detection (two classes: low, high)	ECG, RESP, and skin TEMP	Modified U-Net with continuity-aware loss function	Accuracy: 80%
Wang et al. (2019) [291]	Automotive	Real-world driving	Driving stress levels (low, high)	ECG and GSR	CNN	Accuracy: 92%, specificity: 92%, sensitivity: 93%, AUC: 98% (the average evaluating metrics on 10 drivers)
Huang et al. (2020) [284]	Automotive safety	Simulated driving scenarios	Driver cognitive stress levels (low, normal, high)	ECG (converted into pictures)	CNN	Accuracy: 92.8% (28 interbeat intervals), 98.79% (40 interbeat intervals)
Lingelbach et al. (2021) [292]	Drivers’ stress recognition	Simulated driving with cognitive tasks	Three cognitive stress levels (low, mid, high)	EDA	Conventional ML, AutoML, CNN	CNN was not superior in performance compared to the conventional and AutoML models with handcrafted features
Bustos et al. (2021) [283]	Driver assistance technologies	Real-world driving	Stress levels (low, medium, high)	Road scene video data	CNN, TSN (Temporal Segment Networks)	Best accuracy of the TSN model: 0.72
Tzevelekakis et al. (2021) [293]	Driver’s cognitive load estimation	Real-world driving (DriveDB dataset)	Mental stress classification: two-class (stress vs. non-stress) and three-class (low, moderate, high)	ECG	VGG-inspired model and 1D-CNN model	Accuracy: 98.3% for two-class classification (VGG-inspired model), 85.1% for three-class classification (1D-CNN model)
He et al. (2022) [294]	Driver’s cognitive load estimation	Simulated driving with an auditory–verbal n-back task	Three-level of driver’s cognitive load (no external load, one-back task, two-back task)	Eye-tracking, ECG, and GSR	Conventional ML models and RNN model	Best accuracy of 97.8% achieved by RF model, where the RNN model achieved 95.6%
Mohd Isam et al. (2022) [285]	Drivers’ stress recognition	Real-world driving	Three-level of driver’s stress (low, medium, high)	EMG (converted into 2D spectrogram)	Pre-trained CNNs (SqueezeNet, GoogLeNet, ResNet50)	Best validation accuracy of 66.67% achieved by GoogLeNet-based model
Amin et al. (2022) [286]	Drivers’ stress detection	Real-world driving	Three-level of driver’s stress (low, medium, high)	ECG (converted by Continuous Wavelet Transform)	Pre-trained CNN models (Xception, GoogLeNet, DarkNet-53, ResNet-101, InceptionResNetV2, DenseNet-201, InceptionV3)	Best overall validation accuracy of 98.11% achieved by Xception-based model
Huang et al. (2022) [295]	Recognition of drivers’ mental workload	Simulated driving with a secondary task of various difficulties	Four-class of driver’s mental workload (rest, normal, high, very high)	EEG, ECG, EDA, and RESP	CNN- and LSTM-based models	Highest accuracy with CNN-LSTM: 97.8%
Aygun et al. (2022) [296]	Recognition of drivers’ cognitive workload	Simulated driving with various secondary tasks	Three binary classifications derived from three cognitive workload levels	Pupillometry, EEG, HRV, and BPV	MLP, LSTM	A comprehensive evaluation were conducted on ML models and sensing modalities
Wei et al. (2023) [297]	Forecasting the driver’s mental workload	Real-world driving	Three-level of driver’s mental workload (low, medium, high)	ECG and EDA	LTS-MPF (transformer-based model)	Classification accuracy: 94.3%, future workload prediction (1 s) accuracy: 93.5%
Zontone et al. (2023) [287]	Drivers’ stress detection	Simulated and real-world driving	Two-level of driver’s stress	EDA and ECG (both converted into scalogram)	CNN	Simulated driving: 91.78% accuracy, real driving: 99.24% accuracy
Shajari et al. (2023) [298]	Detection of driver’s cognitive distraction	Simulated driving with cognitive tasks	Cognitive distraction (distracted, not distracted)	Eye-tracking, head movement data, and driving performance measures	Deep feedforward neural network (D-FFNN)	Accuracy: 96.09%, precision: 95.7%, recall: 95.56%, F1-score: 95.63%
Aminosharieh Najafi et al. (2023) [299]	Drivers’ mental engagement analysis	Simulated driving with manual and autonomous scenarios	Mental engagement (high, low)	EEG, EDA, and ECG	Deep CNN with data-/feature-level fusion methods	Average accuracy on LOSO validation: 82.0%
Fenoglio et al. (2023) [268]	Privacy-aware cognitive workload estimation	Semi-autonomous driving simulation experiment with n-back test (ADABase dataset)	Two-level of driver’s cognitive workload (low, high)	Eye-tracking, ECG, EMG, and EDA	Federated learning (FL), CNN	Average accuracy on LOSO validation: 85.58%
Amadori et al. (2023) [300]	Automotive	Simulated virtual reality driving with secondary tasks	Four-class of driver’s cognitive workload	Eye-tracking, head pose, ECG, and EDA	WorkNet ( an end-to-end sequential network based on HyperLSTMs)	F1-score: 90%

#### 5.1.3. Aviation-Related Task

This subsection examines six studies (shown in Table 5), focusing on cognitive state assessments in aviation. These studies utilise simulated flight and air traffic control tasks to induce cognitive load and stress, mirroring real-world challenges faced by aviation professionals. Tasks range from simulated airfield traffic pattern flights to complex flight manoeuvres and air traffic control scenarios, serving as robust tests to evaluate pilot workload, stress levels, and operational error prediction. Such simulations are pivotal for developing and refining DL models aimed at enhancing safety and efficiency in aviation settings.

A variety of modalities are utilised to measure the physiological and performance factors influencing cognitive state assessment in aviation environments. ECG, featured in three studies, provides insights into heart activity. Pupilometry, also utilised in three studies, tracks changes in gaze, eyelid movement, and pupil size. EDA, featured in three studies, measures skin conductance. PPG, used in one study, measures pulse rate variability. TEMP, ACC, RESP, and EMG, each featured in one study, assess body temperature, acceleration, respiration rate, and muscle activity, respectively. Additionally, performance data is considered in two studies, offering insights into the overall pilot performance. Head pose and facial expression, each assessed in one study, further enhance the understanding of pilot engagement and emotional state. Together, these modalities form a comprehensive set for analysing a wide range of physiological responses and performance metrics, crucial for enhancing aviation safety and efficiency through improved cognitive state recognition.

DL techniques in aviation cognitive state assessment encompass traditional models like DNNs, CNNs, and RNNs, along with more advanced architectures such as CNN-transformers hybrids, aiming to integrate spatial and sequential learning capabilities for enhanced data analysis. For instance, Li et al. employ CNN to extract features from concatenated hand-crafted vectors and enhance analysis with the transformer’s attention mechanism [301].

**Table 5:** Table of studies on aviation-related tasks using multimodal physiological modalities and deep learning (2010–2023).

Authors	Practical Application	Practical Task	Classification Goal and Classes	Key Features and Modalities	Deep Learning Approach	Classification Performance
Wang et al. (2020) [302]	Pilot workload evaluation	Simulated airfield traffic pattern flights	Cognitive load levels: low, medium, high	ECG	LSTM-RNN hybrid model	Accuracy 77.6%
Meneses et al. (2021) [303]	Pilot stress monitoring system	Simulated stress-inducing tasks	Stress detection (stressed vs. non-stressed)	PPG, EDA, TEMP, and ACC	Deep convolutional neural network (DCNN)	Binary classification accuracy: 98% This DCNN model has also been validated on other datasets. Accuracy: 99% (WESAD), 90% (NASA-TLX calibration), 87% (ELWEV)
Zhang et al. (2023) [304]	Pilot workload evaluation	Simulated flight tasks	Classification of three levels of pilot workload	Eye-tracking and performance data	Multi-population genetic backpropagation neural networks	Faster convergence and lower prediction error than genetic backpropagation neural networks
Moore et al. (2023) [19]	Pilot cognitive load monitoring	Simulated flight tasks	Classification of pilot task difficulty (four levels)	Eye-tracking and EDA	DNN with MINIROCKET for feature reduction	AUC: 0.912; equal error rate: 0.181
Li et al. (2023) [301]	Pilot stress monitoring	Flight simulation maneuvers	Stress level classification (two, three, and four classes)	ECG, EMG, EDA, RESP, and TEMP	Transformer with CNN	Accuracy: 93.28% (two-class), 88.75% (three-class), 84.85% (four-class)
Xiong et al. (2023) [305]	Air traffic control	Simulated air traffic control tasks	Prediction of separation errors (binary: error/no error)	Head pose, eyelid movement, facial expression, ECG, and task performance measures	Encoder–decoder LSTM network	Precision, recall, F1-score, alignment accuracy, sequence similarity

#### 5.1.4. Medical Task

Table 6 presents studies by Sharma et al. [306] and Jin et al. [9], which explore cognitive state assessment in medical environments, focusing on different settings and methodologies. Sharma et al. address cognitive workload during fetal ultrasound examinations using pupil diameter changes as a physiological indicator, employing 1D-CNN and 2D-CNN (ResNet18) models to classify cognitive workload. Jin et al. assess cognitive workload in a surgical setting, specifically during laparoscopic peg transfer tasks under four distinct conditions. They use a combination of EEG, fNIRS, and pupil diameter data, analysed through a multimodal DL approach incorporating transfer learning with AlexNet and other CNNs. Notably, both studies effectively utilised CNNs and pre-trained models. This approach allows for enhanced feature extraction and efficient processing of complex data, reducing the training time and computational resources required.

#### 5.1.5. Virtual Reality

In the context of virtual reality (VR) applications, two studies focus on immersive experiences to assess cognitive states. Amadori et al. utilise simulated driving setups in VR to detect cognitive overload during the N-back task. Ahmad et al. assess stress levels using a VR rollercoaster simulation. Notably, similar to the approach seen in studies analysed previously, Amadori et al. utilise ECG signals by converting them into spectrograms. These spectrograms are then combined with raw ECG signals and fed into a weighted average fusion CNN model. Indeed, this approach follows the intuition behind CNNs’ ability to extract spatial features from spectrogram images, enhancing the modelling of physiological data. Table 7 provides more detail regarding the evaluation of these two VR studies, demonstrating the applicability of physiological signals in immersive VR environments for assessing cognitive states.

#### 5.1.6. Working Environment

In the working environment sector, specifically within manufacturing (shown in Table 8), Donati et al. investigate worker well-being by leveraging ECG data with a 1D-CNN to detect stress, achieving an accuracy of 88.4% and an F1-score of 0.90. Their findings underline the significant interplay between worker health and productivity, suggesting that monitoring stress can be important to maintaining production quality and overall efficiency in the workplace.

### 5.2. Advancements in Multimodal Physiological Data and Deep Learning for Real-World Cognitive State Recognition

This subsection synthesises findings from an extensive analysis of multimodal physiological modalities and DL techniques across various sectors, encompassing 55 studies in total, highlighting key advancements in real-world cognitive state recognition. ECG is identified as the most frequently used modality, featured in more than half of the studies—specifically, 12 in cognitive load-inducing tasks, 14 in driving tasks, 3 in aviation-based tasks, 1 in VR, and 1 in manufacturing. Either raw signal data or derived features, such as HRV and heart rate, are recognised as reliable indicators in the assessment of cognitive states, making them valuable for DL analyses in real-world applications.

**Table 6:** Table of studies on medical tasks using multimodal physiological modalities and deep learning (2010–2023).

Authors	Practical Application	Practical Task	Classification Goal and Classes	Key Features and Modalities	Deep Learning Approach	Classification Performance
Sharma et al. (2021) [306]	Cognitive workload assessment in clinical ultrasound imaging	Fetal ultrasound examinations	Cognitive workload classification	Pupil diameter changes	1D-CNN, 2D-CNN (ResNet18)	ROC AUC: 0.98 (task), 0.80 (experience)
Jin et al. (2022) [9]	Cognitive workload assessment in surgical settings	Laparoscopic peg transfer in four distinct conditions	Cognitive workload (binary and four-class)	EEG, fNIRS, and pupil diameter	Multimodal DL with transfer learning (AlexNet) and CNNs	Accuracy: 100% (binary), 93% (four-class)

**Table 7:** Table of studies on virtual reality tasks using multimodal physiological modalities and deep learning (2010–2023).

Authors	Practical Application	Practical Task	Classification Goal and Classes	Key Features and Modalities	Deep Learning Approach	Classification Performance
Amadori et al. (2020) [307]	Simulated driving setups using VR	Simulated driving while performing N-back task	Cognitive overload detection; binary classification of decision correctness	Eye-gaze and head-pose data	LSTM-based model (DecNet)	Cognitive overload can be detected 2 s before it occurs in both auditory stimuli (81% precision, 77% recall) and visual stimuli (67% precision, 65% recall)
Ahmad et al. (2023) [308]	Stress assessment in VR applications	VR rollercoaster simulation	Stress level classification into low, medium, high (three classes)	ECG	Multimodal deep fusion model (CNN based)	Outperformed classical models and baseline DL models, showing a 9% increase in accuracy over HRV-based ML models and a 2.5% increase over baseline DL models

**Table 8:** Table of studies on manufacturing using multimodal physiological modalities and deep learning (2010–2023).

Authors	Practical Application	Practical Task	Classification Goal and Classes	Key Features and Modalities	Deep Learning Approach	Classification Performance
Donati et al. (2023) [309]	Worker stress detection in manufacturing sectors	Workers producing in factory	Binary classification of stress conditions (stress vs. not stress)	ECG	1D-CNN	Accuracy: 88.4%, F1-score: 0.90

CNNs are found as the most frequently used DL approaches, either solely or in hybrid models, in more than half of the studies (34 out of 55). The distribution includes 15 in cognitive load-inducing tasks, 13 in driving tasks, 2 in aviation-based tasks, 2 in medical tasks, 1 in VR, and 1 in manufacturing. Three main reasons account for their prevalence: firstly, CNNs are adept at interpreting images, which is crucial when time series data such as ECG and EMG are converted into image-like formats or when the study involves image or video data. Secondly, they can effectively leverage pre-trained CNNs such as AlexNet and ResNet, enhancing their efficiency without the need for extensive training from scratch. Lastly, they serve as robust feature extraction mechanisms, often combined with LSTM and transformer models to handle long-term dependencies and attention mechanisms (9 out of 34 studies).

Indeed, the combination of ECG signals and CNN-based architectures seems to be a promising direction for future research in cognitive state recognition. A significant percentage, 68% (21 out of 31), of studies utilising ECG also employ CNN-based architectures, whether solely CNN or in hybrid forms [4,11,20,268,272,276,278,280,281,282,284,286,287,290,291,293,295,299,301,308,309]. This indicates a strong trend towards integrating these two technologies to enhance accuracy and efficiency in analysing physiological data. Additionally, the growing focus on data security and privacy is facilitating the adoption of privacy-preserving methodologies [265,267,268]. Techniques like DP and FL are increasingly applied to safeguard sensitive physiological data, ensuring compliance with privacy laws and enhancing the feasibility of deploying cognitive state recognition technologies in privacy-sensitive environments.

While these findings highlight significant methodological advancements, certain limitations within the current body of evidence should be acknowledged. A large proportion of studies are concentrated in automotive and cognitive load-inducing task domains, whereas relatively few have been conducted in medical or surgical settings. Moreover, many studies rely on complex or intrusive sensing modalities, such as EEG and fNIRS, which may limit their practical deployment in real-world applications. These gaps suggest a need for broader domain representation and further development of non-intrusive sensing technologies to facilitate more widespread adoption of cognitive state recognition systems.

## 6. Conclusions

This systematic review comprehensively analyses advancements in cognitive state recognition from 2010 to early 2024, evaluating 405 relevant articles selected from an initial pool of 2398 studies. The findings highlight several key trends and developments.

Firstly, there has been a pivotal shift from shallow ML techniques to more sophisticated DL approaches in the analysis of physiological signals. This transition is driven by DL’s ability to autonomously learn and decipher intricate patterns in large datasets, demonstrating a more robust solution for cognitive state recognition. By 2023, DL has become the dominant methodology, although traditional ML techniques continue to maintain their relevance.

Secondly, the review documents a shift from neuroimaging to multimodal physiological modalities in cognitive state recognition, while neuroimaging modalities, especially EEG, have dominated the field (featured in 218 out of 405 articles), they face challenges such as setup complexity and the need for specialised interpretation skills. Recent trends show a decrease in neuroimaging use in multimodal studies, from 19.4% between 2011 and 2020 to 16% in 2023, reflecting a move towards integrating various physiological signals. Despite this, combining physiological and neuroimaging signals remains valuable for comprehensive insights in specific scenarios.

Thirdly, cognitive state recognition has been integrated into a diverse range of domains, all aiming to enhance performance and safety. This broad application spans sectors such as automotive, aviation, maritime, and healthcare, with a notable emphasis on cognitive load-inducing tasks. These applications underscore the field’s impact in professional and everyday settings, particularly in high-stakes environments. Notably, only 10 out of 405 studies are medical, indicating a low adoption in the healthcare sector. One potential limitation to broader implementation in surgery is the obtrusive nature of multiple wearable sensing modalities, which can be intrusive and cumbersome for surgical teams. To address this, initiatives like the MAESTRO project aim to optimise cognitive state recognition frameworks to enhance surgical workflow understanding while minimising disruption to the surgical team. This involves using less intrusive sensors, such as ECG and eye tracking, as practical substitutes for more intrusive options like fNIRS and EEG, maintaining comprehensive monitoring while minimising disruption. Such data-driven surgery approaches advocate for holistic and human-centred sensing methodologies, enabling comprehensive, accurate, and meaningful monitoring that integrates seamlessly into surgical workflows. Furthermore, employing a data-driven surgical approach facilitates improved decision-making, situational awareness, and enhanced patient safety by providing actionable insights directly informed by cognitive state monitoring.

Lastly, the advancements in multimodal physiological data and DL for real-world cognitive state recognition are significant. ECG is the most utilised modality, appearing in a majority of studies across various domains. CNNs are the primary DL approach, used either alone or in hybrid models, across 34 out of 55 studies. The popularity of CNNs is attributed to their effectiveness in image interpretation, utilisation of pre-trained models, and robust feature extraction mechanisms. A promising future research direction is the integration of ECG signals with CNN architectures, with 68% of ECG-utilising studies employing CNNs. Additionally, there is a growing focus on data security, with the adoption of privacy-preserving methodologies like differential privacy and federated learning to protect sensitive data and ensure compliance with privacy regulations. These advancements collectively highlight the transformative potential of cognitive state recognition in enhancing performance, safety, and innovation across diverse real-world applications.

### 6.1. Limitations of the Review Process

This review has several methodological limitations. Although screening was conducted by two independent reviewers, data extraction was performed by a single reviewer and verified by a second, which may introduce a minor risk of bias. Additionally, the review protocol was not registered, as diagnostic and trend-focused reviews were not eligible for PROSPERO registration at the time of review. Risk of bias and certainty assessments were not conducted, given the focus on methodological and application trends rather than on synthesising outcome effect estimates. Similarly, no formal quality appraisal of individual studies was conducted using standardised tools, as the substantial methodological heterogeneity across study designs, physiological signals, and analytical approaches made such an assessment inappropriate. Finally, the synthesis was descriptive in nature and did not include quantitative meta-analysis, which may limit the comparability of performance metrics across studies.

### 6.2. Implications for Future Research and Applications

The findings of this review suggest that future research should prioritise the development of lightweight, non-intrusive sensing systems to improve the usability of cognitive state recognition technologies in real-world settings. Additionally, expanding research across under-represented domains, such as healthcare and collaborative environments, would enhance the generalisability of findings. Although the majority of studies included in this review are situated in non-medical contexts, such as cognitive load-inducing tasks and conventional driving, this distribution reflects the current state of the literature and does not result from selection bias. This imbalance highlights the need for further investigation in surgical and clinical settings, particularly in support of initiatives such as the MAESTRO project, which seeks to improve cognitive state monitoring in high-stakes medical environments. In addition, greater emphasis on privacy-preserving techniques and deployment feasibility will be essential for translating laboratory models into practical, trustworthy solutions across both medical and non-medical domains.

## Figures and Tables

**Table 1:** Comparison of Shallow ML and DL Methods.

Aspect	Shallow ML	DL
Feature engineering	Requires extensive feature engineering. Features must be manually extracted and selected.	Minimal or no feature engineering required. Can automatically learn features from raw data.
Data handling	Better suited for smaller, well-curatead datasets.	Excels with large datasets and can handle high-dimensional data.
Model complexity	Relatively simple models. Easier to interpret and understand.	Complex models with multiple layers. Often considered a `black box’ due to their complexity.
Computational resources	Generally requires less computational power and resources.	Requires significant computational resources for training and processing, especially with large data.
Flexibility	Limited in handling raw data. Dependent on the quality of feature engineering.	Highly flexible in handling various types of raw data.
Interpretability	High interpretability due to simpler models and reliance on handcrafted features.	Lower interpretability due to complex model structures and automatic feature extraction.
Applications	Suitable for applications where interpretability is crucial and data is limited.	Ideal for applications with large datasets and where model complexity can capture intricate patterns.
Temporal data handling	Less effective in capturing temporal dependencies in time-series data.	More effective in processing sequential and time-series data (e.g., using RNNs, LSTMs).

## Data Availability

Not applicable.

## Supplementary Materials

The following supporting information can be downloaded at: https://www.mdpi.com/article/10.3390/s25134207/s1. To ensure transparency and methodological rigour, this review was conducted in accordance with the PRISMA 2020 guidelines. The completed PRISMA 2020 Checklist and the PRISMA 2020 for Abstracts Checklist are provided below as supplementary figures. In addition, Supplementary Table S1 outlines explanatory notes for specific PRISMA items marked as not applicable. Together, these materials support the reproducibility and clarity of the review process and demonstrate compliance with current systematic review reporting standards.
